# Dorsal root ganglion stimulation for treatment of chronic postsurgical pain secondary to triple neurectomy

**DOI:** 10.1016/j.inpm.2023.100245

**Published:** 2023-03-23

**Authors:** Anishinder Parkash, Joe H. Ghorayeb, Isaiah Levy, Aman Upadhyay, Suresh Srinivasan, Gaurav Chauhan

**Affiliations:** a Department of Physical Medicine and Rehabilitation, Tower Health Reading Hospital/Drexel, University COM, 420 S 5th Ave, West Reading, PA, 19611, USA; b University of Medicine and Health Sciences, 275 7th Ave 26th Floor, New York, NY, 10001, USA; c Department of Physical Medicine and Rehabilitation, University of Pittsburgh Medical Center, 200 Lothrop Street, Pittsburgh, PA, 15213, USA; d Department of Anesthesiology and Perioperative Medicine, McLaren Oakland Hospital 50 Perry St, Pontiac, MI, 48342, USA; e Department of Anesthesiology and Perioperative Medicine, University of Pittsburgh Medical Center, 200 Lothrop Street, Pittsburgh, PA, 15213, USA; f Department of Pain Medicine, **4**000 Johnson Rd, Steubenville, OH, 43952, USA

**Keywords:** Inguinal pain, Chronic post-surgical pain, Dorsal root ganglion stimulation, Spinal neuromodulation, Triple neurectomy, Chronic pain syndrome

## Abstract

Triple neurectomy (resection of Ilioinguinal, Iliohypogastric, and Genitofemoral nerves) is performed in cases of inguinal neuralgia, refractory to conservative management. However, this procedure comes with several adverse effects, including but not limited to ectopic afferent firing and tactile allodynia. In such a scenario, the inguinal pain can become chronic and debilitating and can be classified as chronic post-surgical pain. Spinal neuromodulation techniques have been employed for treating such refractory, intractable chronic groin, pelvic and abdominal pain. One such technique is dorsal root ganglion stimulation which is designed to manage difficult-to-treat chronic pain in specific areas of the lower body, such as the foot, knee, hip, or groin. The authors present a case in which the patient underwent a laparoscopic neurectomy of ilioinguinal, Iliohypogastric, and genitofemoral nerves that failed to resolve her pain-related symptoms. The patient presented to the authors’ pain clinic with severe inguinal pain and allodynia, refractory to multiple analgesic agents. The patient underwent a successful trial and subsequent implant with ipsilateral dorsal root ganglion stimulation at L1& L2. At six months post-implant, the patient continues to report 80–90% improvement in her pain and physical function.

## 1 Introduction

Chronic postsurgical pain (CPSP) is defined as pain and associated symptoms that persist more than three months after surgery. CPSP usually differs in quality and location from the pain experiences before surgery and persist for more than three months post-surgery. CPSP is hypothesized to be caused by surgical injury or inflammation to a major peripheral nerve as well as central (spinal and supraspinal) sensitization [1]. A wide range of surgical procedures can cause CPSP at varying incidence rates, with Cesarean section accounting for 15.4% of CPSP cases [1,2].

Globally, 21.1% women gave birth via cesarean section, and it is estimated that this number will rise to 28.5% by 2030 [3]. Borges et al. estimated the risk of developing CPSP following Cesarean section to be 25.5% [4]. The likelihood of developing this painful condition raises a significant concern given the negative impact that CPSP can exert on activities of daily living and quality of life, despite the potential reduction in both maternal and fetal morbidity and mortality [5].

The sensory innervation of the anterior abdominal wall, groin, and pelvic regions is provided by the ilioinguinal (II), Iliohypogastric (IH) and genitofemoral (GF) nerves [6]. The II and IH nerves originate from the anterior rami of the L1 nerve roots with contributions from either T12 or L2, emerging near the lateral border of the psoas major muscle and extending diagonally toward the iliac crest. The GF nerve tends to originate predominantly from L1 and L2, and after the intrapelvic course, it enters the abdominal wall at the level of the deep inguinal ring [6,7]. Neuropathic pain in the regions served by II, IH and GN nerves can be instigated, post-surgically, by compression due to fibrosis or suture materials or when they are partially or completely transected [1,8]

If the symptoms of neuropathic pain following a Cesarean section do not resolve over time, conservative care is initially recommended in the form of lifestyle modification, analgesic use, and nerve blocks [8]. If CPSP develops following Cesarean section, the current consensus favors resection of all three nerves, referred to as a triple neurectomy [9]. Though this procedure may be helpful, it is not bereft of risks, including ectopic afferent firing and tactile allodynia in the inguinal region [10,11]. In such cases of inguinal neuralgia, refractory to surgical resection nerves, neuromodulation techniques, such as dorsal root ganglion stimulation (DRGS) therapy, can be considered [12,13]. The authors report the successful use of DRGS therapy for CPSP of the left inguinal region post-cesarean section, refractory to conventional medical management and worsened after triple neurectomy.

## 2 Clinical vignette

A 36-year-old woman, who consented to this case report, presented to the pain clinic with more than six months of constant burning and lancinating in the left lower abdominal compartment and groin. The patient reported that the pain started after a cesarean section and changed character over four months from sharp, localized discomfort to burning, pins and needles-like paresthesia to knife-like cutting pain. The patient localized her pain along the left inguinal region, radiating above and below the surgical scar to the lower abdomen and the labia majora. The inguinal pain worsened with activity and was associated with painful micturition and defecation. Five months after cesarean section and after multiple treatment failures (including gabapentin and topical compound creams) patient underwent nerve block in the lower left abdominal compartment for ilioinguinal, Iliohypogastric, and genitofemoral nerve block (10 ​cc of 0.5% bupivacaine with 10 mg of dexamethasone). The nerve block provided symptom mitigation only for 3–4 days. Subsequently, the patient underwent surgical resection of II, IH, and GN nerves on the left side at an outside facility. The patient reported no resolution of her pain and associated symptoms after surgery.

**Fig. 1:**
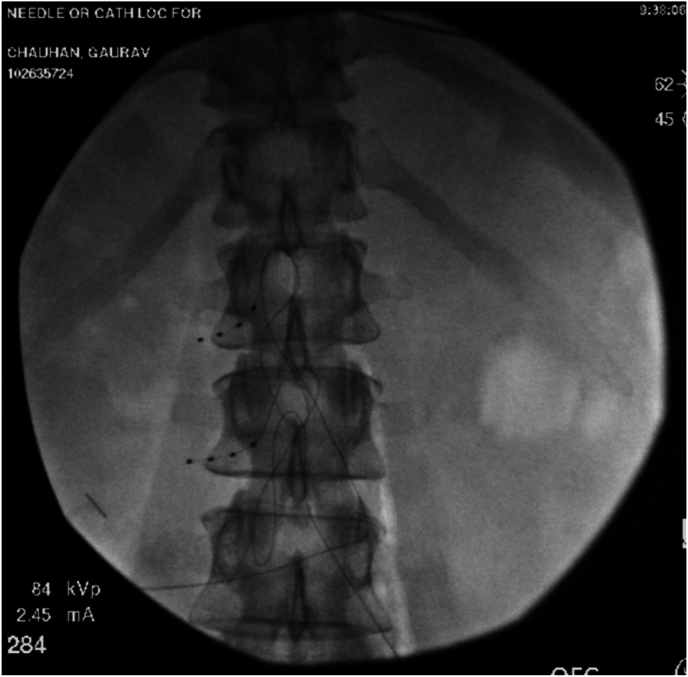
Anteroposterior fluoroscopic view of L1, L2 neuroforaminal dorsal root ganglion lead placement.

**Fig. 2:**
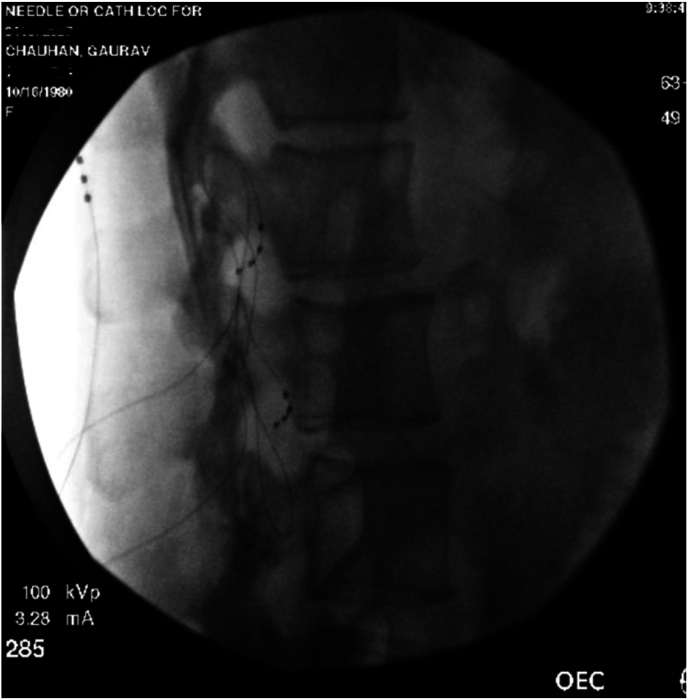
Lateral fluoroscopic view of L1, L2 neuroforaminal dorsal root ganglion lead placement.

**Fig. 3:**
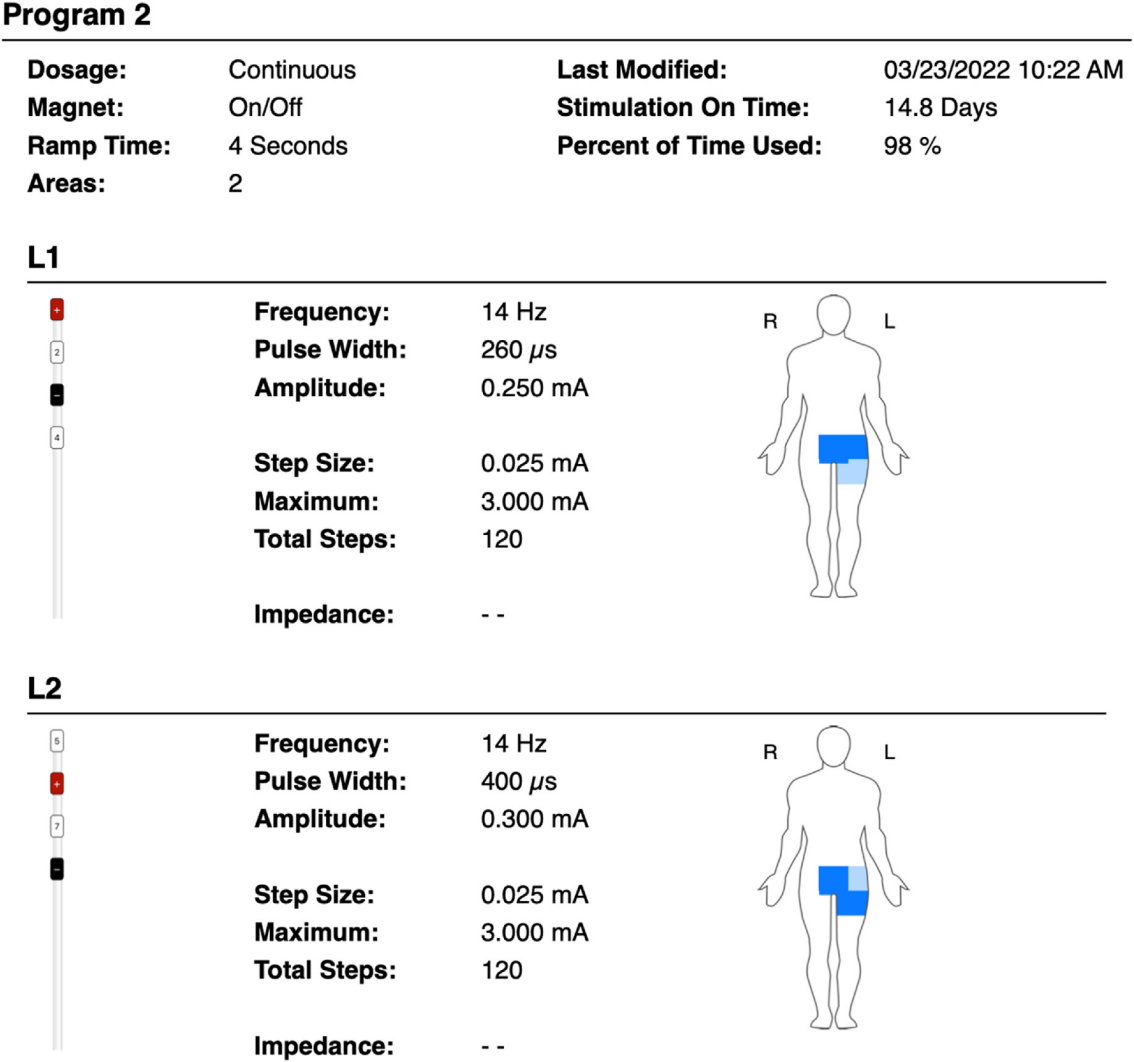
Permanent stimulation parameters for the DRGS L1, L2 leads.

The patient presented to the authors’ clinic three months after her neurectomy surgery and reported worsening her symptoms post-surgery. The patient reported that pain is now 8–10/10 on the numeric rating scale, worsened upon the activities like walking, getting up from a sitting position, and during acts of defection and micturition. The patient also had severe limitations of outdoor activities. The patient also reported that the pain is affecting her sleep. The patient reported a slight alleviation of pain upon taking anti-neuropathic agents. The patient was currently on hydromorphone 4 mg three times daily as needed for severe pain, pregabalin 200 mg three times daily, lidocaine 4% patches to be applied topically over the affected area (12 hours on, 12 hours off), and nortriptyline 75 mg once daily. Pulsed radiofrequency is not covered under insurance at the author’s institution. Therefore, the authors had no other option but to provide a solution proximal to the point of entry of afferent pain fibers. The patient was advised and counseled regarding dorsal root ganglion stimulation (DRGS) therapy. The patient underwent a DRGS trial with leads placed in the left L1 and L2 foramen (Fig. 1, Fig. 2). The leads were programmed to provide adequate coverage of the painful areas. The patient reported 80–90% resolution of her pain and symptoms over the ten-day trial period. The patient underwent an uneventful DRGS implant three weeks after the trial (Fig. 3). The patient underwent successful programming of the leads (Fig. 3) post-implant. The patient reported a significant reduction of pain and was weaned off hydromorphone and pregabalin over 1-month post-implant. At eight months post-DRGS implant, the patient reports 90% resolution of her symptoms. The patient is currently using Tylenol 650 mg and ibuprofen 800 mg for breakthrough pain.

## 3 Discussion

Spinal neuromodulation has proved to be an effective strategy in managing several chronic pain disorders and improving functional outcomes, enhancing the quality of life, and reducing the burden of analgesic medication [14,15]. One of the main challenges faced with treating pain in the lower abdominal walls, groin, and pelvis with neuromodulation is complex nerve innervation. Conventional dorsal column spinal cord stimulation is limited in its ability to target focal dermatomal areas and discrete anatomical regions. In such scenarios, DRGS may be an attractive option due to its increased accuracy concerning target stimulation [[16], [17], [18]].

The dorsal root ganglion (DRG), located in the intervertebral foramen, receives afferent nociceptive input from primary sensory neurons in the periphery. DRG is hypothesized to play a vital role in processing, modulating, and maintaining chronic pain signals [19]. DRGS is a spinal neuromodulation technique designed to target DRG neurons and is usually considered superior to conventional treatment for difficult-to-treat pain diagnoses such as complex regional pain syndrome [20]. While the precise mechanism(s) of DRGS remains to be elucidated, it is hypothesized that DRGS suppresses neuropathic pain by decreasing the neuronal excitability of the DRG cells [19,21].

The bulk of the literature to date regarding the efficacy of DRGS pertains to the treatment of complex regional pain syndrome (CRPS); a rare and debilitating pain disorder that affects one or more limbs with a reported worldwide prevalence ranging from 5.5% to 26.2% per 100,000 people per year [22,23]. CRPS is distinguished based on the absence (type I, accounting for 90% of cases) or presence (type II) of peripheral nerve lesions [24]. The ACCURATE study [25], a prospective, randomized, controlled, multicenter study to evaluate the safety and efficacy of the DRGS compared to traditional SCS for individuals with CRPS or causalgia demonstrated that patients who underwent a permanent DRGS device implantation achieved statistically superior treatment success (defined as 50% reduction in the visual analog scale (VAS) score)

when compared to individuals treated with low-frequency tonic SCS at 3-, 6-, 9-, and 12-months post-implant. Similarly, a single center, retrospective study by Verrills et al. [26] found that DRGS provided paresthesia-free pain relief in 87.2% of patients (N ​= ​34/39) with intractable neuropathic pain at all time points assessed (3-, 6-, and 12-months post-implant).

Current literature offers promise that DRGS is an effective analgesic option for patients with chronic pain in the lower abdomen, groin, and pelvic regions [[21], [22], [23]]. Schu et al. reported that DRGS was effective at managing chronic pelvic pain arising from multiple etiologies, with 82.6% of patients (N ​= ​19/23) reporting a >50% reduction in pain [11]. In Morgalla et al.'s study, the patients with persistent inguinal pain post herniorrhaphy experienced 63.5% ​± ​10% pain relief after 3 years [12]. Several case reports and case series have also reported successful outcomes with DRGS treating chronic pelvic pain of multiple etiologies with sustained reductions in pain over follow-up periods as short as two months to three years [[21], [22], [23]]. The authors unanimously agree that DRGS therapy is a valuable tool in the armamentarium of the chronic pain physician in cases of inguinal neuralgia refractory to conventional medical and surgical modalities.

## 4 Conclusion

Inguinal neuralgia is a common complication of lower abdominal surgeries and is classified under chronic postsurgical pain. Conservative strategies, such as lifestyle modification, pharmacotherapy, and nerve blocks, are often recommended as first-line care. In cases refractory to conventional treatment, pain and other symptoms associated with inguinal neuralgia can be severely debilitating. This report will add to the evidence regarding the successful use of DRGS therapy for chronic postsurgical inguinal pain after cesarean section, which was refractory to conventional medical management and surgical techniques (including triple neurectomy). Larger, randomized studies in this patient population might reveal the actual efficacy of DRGS therapy.

## Declaration of competing interest

The authors declare that they have no known competing financial interests or personal relationships that could have appeared to influence the work reported in this paper.
